# Functional Characterization of Four Putative δ^1^-Pyrroline-5-Carboxylate Reductases from *Bacillus subtilis*

**DOI:** 10.3389/fmicb.2017.01442

**Published:** 2017-08-02

**Authors:** Giuseppe Forlani, Boguslaw Nocek, Srinivas Chakravarthy, Andrzej Joachimiak

**Affiliations:** ^1^Department of Life Science and Biotechnology, University of Ferrara Ferrara, Italy; ^2^Center for Structural Genomics of Infectious Diseases, University of Chicago Chicago, IL, United States; ^3^Argonne National Laboratory, BioCAT, Center for Synchrotron Radiation Research and Instrumentation Argonne, IL, United States; ^4^Department of Biological and Chemical Sciences, Illinois Institute of Technology Chicago, IL, United States

**Keywords:** proline synthesis, P5C reductase, *Bacillus subtilis*, isoenzyme properties, substrate ambiguity, product inhibition, oligomeric structure

## Abstract

In most living organisms, the amino acid proline is synthesized starting from both glutamate and ornithine. In prokaryotes, in the absence of an ornithine cyclodeaminase that has been identified to date only in a small number of soil and plant bacteria, these pathways share the last step, the reduction of δ^1^-pyrroline-5-carboxylate (P5C) catalyzed by P5C reductase (EC 1.5.1.2). In several species, multiple forms of P5C reductase have been reported, possibly reflecting the dual function of proline. Aside from its common role as a building block of proteins, proline is indeed also involved in the cellular response to osmotic and oxidative stress conditions. Genome analysis of *Bacillus subtilis* identifies the presence of four genes (*ProH, ProI, ProG*, and *ComER*) that, based on bioinformatic and phylogenic studies, were defined as respectively coding a putative P5C reductase. Here we describe the cloning, heterologous expression, functional analysis and small-angle X-ray scattering studies of the four affinity-purified proteins. Results showed that two of them, namely ProI and ComER, lost their catalytic efficiency or underwent subfunctionalization. In the case of ComER, this could be likely explained by the loss of the ability to form a dimer, which has been previously shown to be an essential structural feature of the catalytically active P5C reductase. The properties of the two active enzymes are consistent with a constitutive role for ProG, and suggest that *ProH* expression may be beneficial to satisfy an increased need for proline.

## Introduction

**Figure 1:**
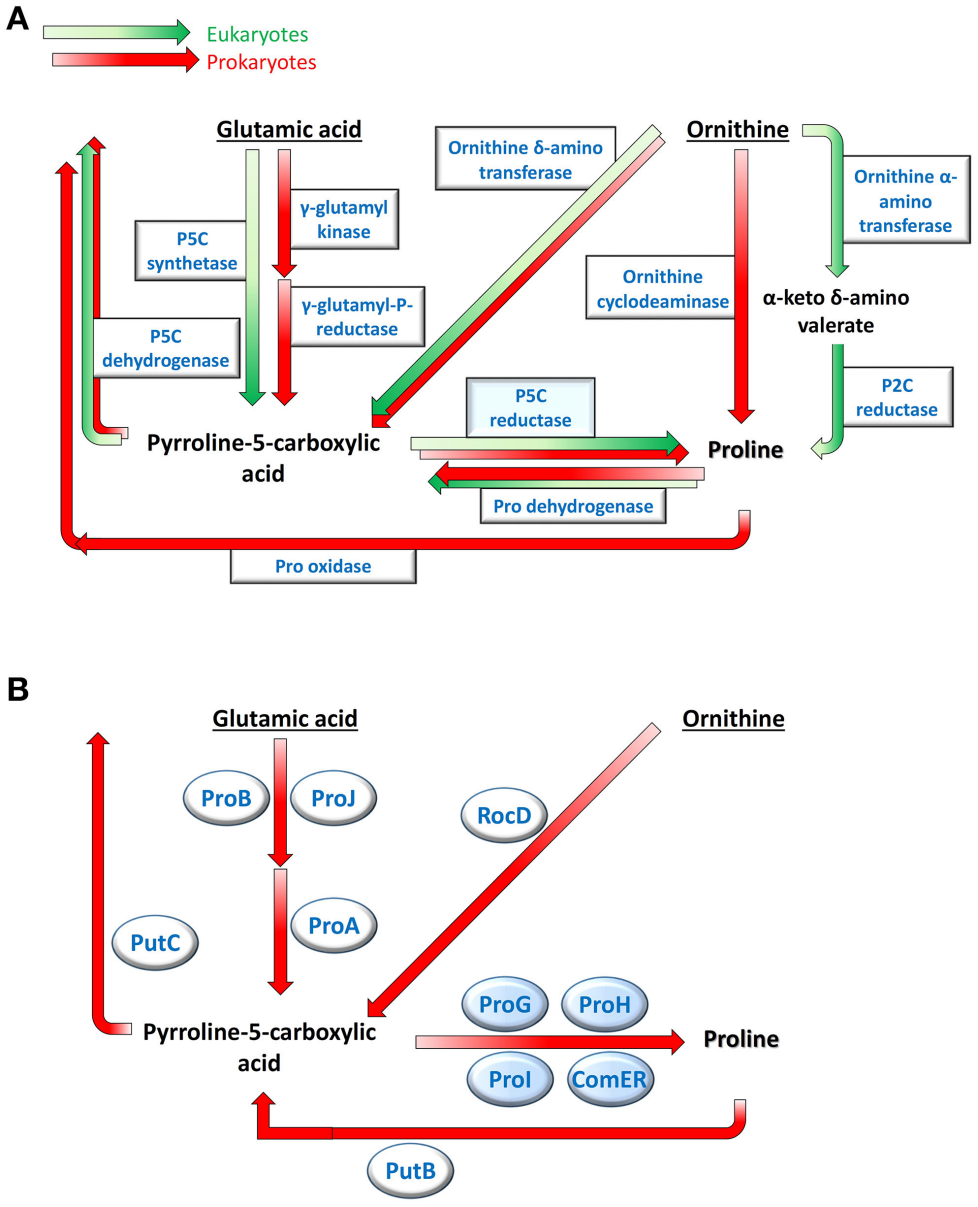
Multiple pathways for proline metabolism. Several routes leading to the production and the re-oxidation of the imino acid have been described in plants and bacteria **(A)**. In *Bacillus subtilis* only two biosynthetic pathways appear to be present, sharing the last step catalyzed by P5C reductase **(B)**. Genes products potentially involved in this Gram-positive bacterium, which were identified by sequence homology, are shown.

Several metabolic routes have been shown to lead to the biosynthesis of the proteinogenic amino acid proline (Figure 1A; Fichman et al., 2015). In higher plants, the primary pathway starts from glutamate, which is converted to δ^1^-pyrroline-5-carboxylate (P5C) by a bifunctional P5C synthetase (Rai and Penna, 2013). In bacteria, the same reaction is accomplished by two enzymes, a γ-glutamyl kinase that catalyzes glutamate phosphorylation and a γ-glutamyl phosphate reductase that reduces the product to glutamate semialdehyde, which in solution spontaneously cyclizes to P5C (Chen et al., 2006; Csonka and Leisinger, 2007). In both cases, the latter is reduced to proline by an enzyme, P5C reductase (Forlani et al., 2015c). Alternatively, ornithine may function as the precursor, being a pyridoxal phosphate-dependent ornithine δ-aminotransferase (δOAT) able to convert ornithine to P5C (Zaprasis et al., 2014; Winter et al., 2015). Also in this case, the last step is then catalyzed by P5C reductase. Being required in both routes, null mutants of P5C reductase have been found embryo-lethal (Funck et al., 2012), and specific inhibitors of the enzyme exert cytotoxic effects in plants (Forlani et al., 2013) and bacteria (Forlani et al., 2012). Although not conclusively proven, in some plant species ornithine could be deaminated instead by an α-aminotransferase (αOAT) yielding α-keto-δ-aminovalerate (Fichman et al., 2015), which is in spontaneous equilibrium with pyrroline-2-carboxylate (P2C). The latter might be reduced to proline by a P2C reductase (Goto et al., 2005). Finally, in a small group of soil and plant-associated bacteria, ornithine can be directly converted to proline by an ornithine cyclodeaminase (OCD) (Jensen and Wendisch, 2013). A gene coding for a putative OCD has been found also in plants, but the ability of the gene product to catalyze proline synthesis has not yet been demonstrated (Sharma et al., 2013).

Such a variety in proline biosynthesis may be based on the multiple roles played by the amino acid in cell metabolism. In fact, besides providing building blocks for protein synthesis, the accumulation of high intracellular levels of this imino acid represents an effective response to a broad range of stress conditions (Szabados and Savouré, 2010). As a compatible osmolyte, proline may counteract the decrease of the external water potential due to drought, salt excess or ice formation, thereby avoiding cell dehydration (Kempf and Bremer, 1998; Takagi, 2008; Bhaskara et al., 2015). Moreover, the presence of the imino acid at millimolar concentrations seems to protect cellular membranes and stabilize the quaternary structure of proteins (Ignatova and Gierasch, 2006). The interconversion of glutamate and proline can regulate (and be regulated by) the NAD(P)H/NAD(P)^+^ ratio (Sharma et al., 2011), and serve as a redox shuttle among cell compartments (Phang et al., 2010). The NAD(H)/NADP(H) ratio can influence in turn the rate of proline synthesis, possibly enhancing its production under stress (Giberti et al., 2014; Shinde et al., 2016) Finally, proline may represent itself a reactive oxygen species (ROS) scavenger (Signorelli et al., 2015), or a signal triggering ROS production leading either to the hypersensitive response to pathogens attack in higher plants (Ben Rejeb et al., 2014), or to apoptosis and tumor suppression in animals (Liang et al., 2013). In this intricate picture, the homeostasis of P5C seems to be of fundamental importance (Qamar et al., 2015), as it lays at the intersection of most biosynthetic pathways and represents an intermediate in both anabolic and catabolic routes (Figure 1A).

Consistently, in spite of catalyzing the last and non-limiting (Kesari et al., 2012) step in these pathways, P5C reductase has been found to be subjected to fine regulation at either the transcriptional (Hua et al., 1997), the translational (Hua et al., 2001) and the post-translational level (Giberti et al., 2014). The enzyme can use NADH or NADPH as the electron donor (Fichman et al., 2015), but recent studies suggest that only the latter is used *in vivo* (Petrollino and Forlani, 2012; Ruszkowski et al., 2015). Interestingly, it has been shown that the NADPH-driven reaction, but not the NADH-dependent one, is strongly stimulated by NaCl concentrations in the 10^−2^–10^−1^ M range, thereby providing a mechanism for rapidly enhancing proline synthesis under osmotic stress conditions without the need of transcriptional control (Giberti et al., 2014; Forlani et al., 2015b). The occurrence of multiple enzyme forms of P5C reductase has been reported in a few plants (Chilson et al., 1991; Murahama et al., 2001) and microorganisms (Belitsky et al., 2001) In human tissues, the presence of a non-allosterically regulated isozyme restricted to erythrocytes, and another ubiquitous and proline-sensitive enzyme form led to hypothesize that the former plays a role in NADP^+^ generation and not in proline synthesis (Merrill et al., 1989).

A recent survey of the over 37,000 genes that by sequence homology have been annotated as putative P5C reductases in the NCBI database pointed out that most species possess only one gene or have lineage-specific duplications (Forlani et al., 2015c). In several bacteria and archaea, however, distant and relatively fast-evolving paralogs have been found (Forlani et al., 2015c) that may represent potential examples of subfunctionalization (Fichman et al., 2015). Yet, in a vast majority of cases the products of these isogenes have not been well-characterized.

**Figure 2:**
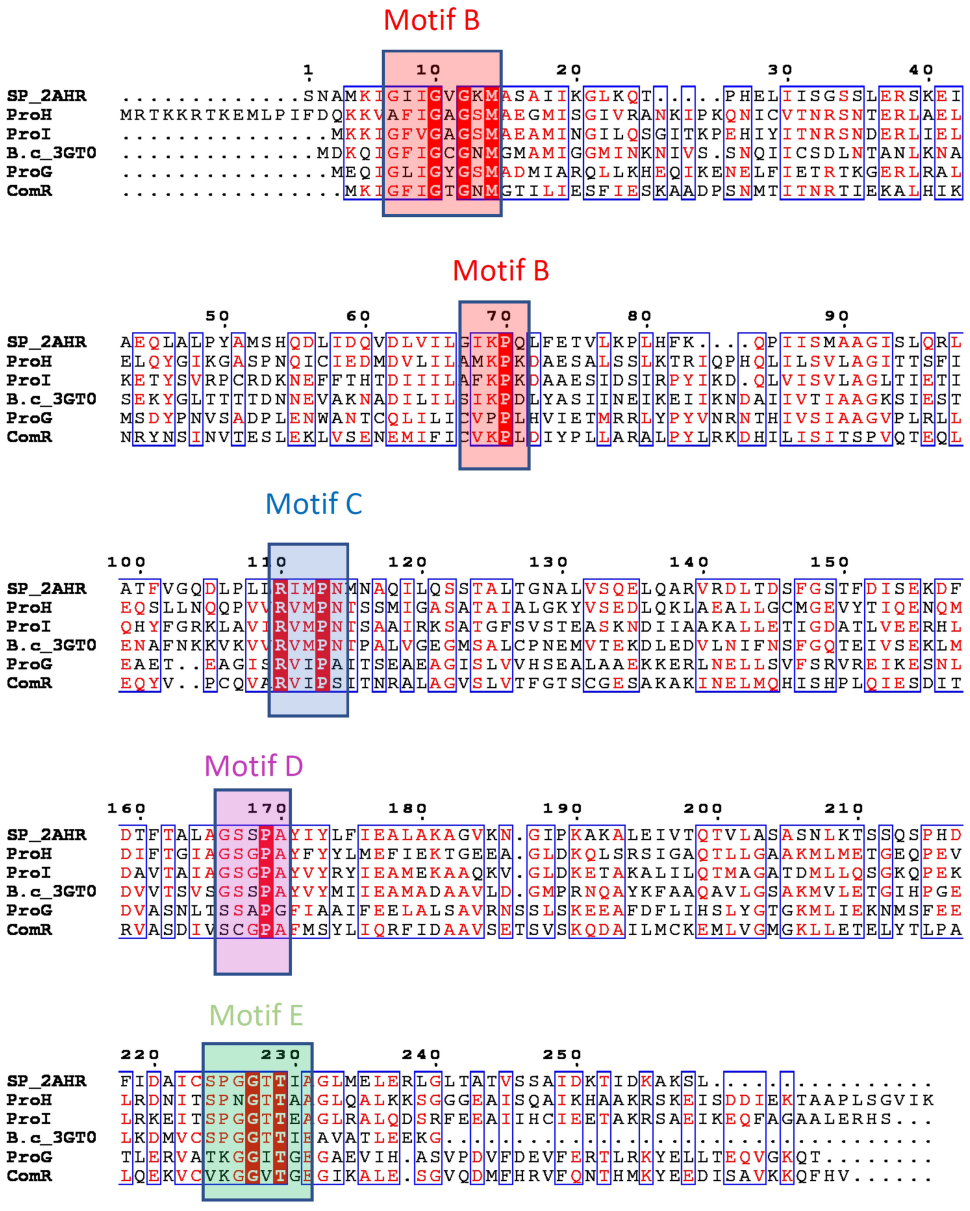
Putative P5C reductases in *Bacillus subtilis* and their bacterial orthologues. Genome sequence analysis points out the presence of four genes that have been predicted by homology as possible P5C reductases (ProG-Q00777, ProH-P0CI77, ProI-P54552, ComER-P39696). Sequences of structurally characterized bacterial members of P5C reductases were added for comparison and to help visualization of the presence of structurally defined motifs (SP_2AHR stands for *Streptococcus pyogenes* PDB id 2AHR, while B.c_3GT0 represents P5C reductase from *Bacillus cereus*, PDB code 3GT0). Deduced protein sequences were aligned using Multalin and rendered in ESPript 3.0 (http://espript.ibcp.fr; Robert and Gouet, 2014). Sequence identities are highlighted in red and similarities are displayed as red letters. The conserved sequence motifs are colored and labeled according to the nomenclature introduced in previous studies (Forlani et al., 2015c; motif B in red, motif C in blue, motif D in magenta and motif E in green).

When exposed to high osmolarity, *Bacillus subtilis* synthesizes large amounts of proline to maintain proper levels of hydration and turgor (Hoffmann et al., 2012). Proline is believed the only compatible osmolyte that is produced *de novo* in this species (Kuhlmann and Bremer, 2002; Zaprasis et al., 2015). Because an *OCD* gene appears absent, proline biosynthesis in *B. subtilis* proceeds *via* P5C (Figure 1B). Mutation analysis showing abolished osmoadaptive proline production as a consequence of the disruption of *proJ, proA*, or *proH* genes clearly demonstrated a prevalence of the glutamate pathway (Brill et al., 2011), whereas ornithine conversion to P5C by RocD -a δOAT- seems to play a role restricted to arginine utilization (Lu, 2006; Zaprasis et al., 2014). Besides *proJ*, which is part of the *proHJ* operon, a second gene coding for a glutamate kinase (*proB*, contained in the *proBA* operon) has been described in *Bacillus* spp. (Zaprasis et al., 2013), yet their respective role in proline synthesis is still unclear. Interestingly, the *B. subtilis* genome also includes no less than 4 genes (namely *proG, proH, proI*, and *comER*; Figure 2) with the potential to encode a P5C reductase. The functionality of the first three gene products was inferred from the fact that their simultaneous defect was required to confer proline auxotrophy (Belitsky et al., 2001). On the contrary *comER*, which is contained with an unusual overlapping and divergent orientation in the *comE* late competence operon (Hahn et al., 1993; Ogura and Tanaka, 2009), is believed not to be involved in proline synthesis (Fichman et al., 2015) since its disruption did not influence either the accumulation of proline (Belitsky et al., 2001) or the acquisition of competence (Inamine and Dubnau, 1995). Recent results suggested a role for *comER* in biofilm formation and sporulation (Yan et al., 2016). However, to date, the ability of these proteins to catalyze P5C reduction has not been assessed, nor the specific role in cell metabolism of active isozymes has been fully elucidated.

To address this question, we cloned the four genes from *B. subtilis* and expressed them in *Escherichia coli*. A detailed biochemical characterization of the affinity-purified proteins allowed us to separate fully active enzymes from those showing only very low catalytic levels with P5C as the substrate, as well as to hypothesize distinct roles for the former in the bacterial cell metabolism.

## Materials and methods

### Cloning and heterologous expression

The sequences coding for the four putative P5C reductase proteins from *Bacillus subtilis* strain 168 were amplified by PCR using the primers reported in Table 1, and cloned into vector pMCSG68 according to the standard protocol described previously (Eschenfeldt et al., 2013; Forlani et al., 2015b). The pMCSG68 vector introduces a His_6_-tag followed by the Tobacco Etch Virus (TEV) protease cleavage site at the N-terminus of the expressed protein. The correctness of the insert was confirmed by DNA sequencing. Overexpression was carried out in BL21 Gold *E. coli* cells (Agilent Technologies). The bacteria were cultured with shaking at 210 rpm in LB medium supplemented with 150 μg mL^−1^ ampicillin at 37°C until the OD_600_ reached 1.0. The temperature was lowered to 18°C, and isopropyl-D-thiogalactopyranoside (IPTG) was added to a final concentration of 0.5 mM. The culture was grown for 18 h and then centrifuged at 4,000 g for 10 min at 4°C. The cell pellet from 1 L culture was resuspended in 35 mL of lysis buffer (50 mM HEPES sodium salt pH 8.0, 500 mM NaCl, 5% glycerol, 20 mM imidazole, 10 mM β-mercaptoethanol) and stored at −80°C.

**Table 1:** Primers used to clone the four putative P5C reductases of *B. subtilis*.

**Gene**	**Primer sequence**
*proH*	Forward 5′– TACTTCCAATCCAATGCCATGCGAACAAAAAAGCGAACAAAGGAGAT –3′
	Reverse 5′– TTATCCACTTCCAATGTTAAGCTTGCAGCCCGGCAGCA –3′
*proI*	Forward 5′– TACTTCCAATCCAATGCCATGAAAAAGATAGGATTTGTCGGCGCC –3′
	Reverse 5′– TTATCCACTTCCAATGTTAAGAATGTCTCTCTAAGGCGGCAC –3′
*proG*	Forward 5′– TACTTCCAATCCAATGCCATGGAACAGATTGGATTGATTGGATATGG –3′
	Reverse 5′- TTATCCACTTCCAATGTTATGTTTGTTTACCAACCTGTTCTGTAAGCA –3′
*comER*	Forward 5′– TACTTCCAATCCAATGCCATGAAGATAGGCTTTATCGGCACAGGAA –3′
	Reverse 5′– TTATCCACTTCCAATGTTACACATGAAACTGCTTTTTCACTGCGGAA –3′

### Protein purification

All proteins were purified according to the standard protocol for Ni-NTA affinity chromatography, as described previously (Forlani et al., 2015c). The His_6_-tag was removed by treating each enzyme with His_6_-tagged TEV protease for 16 h at 4°C in 50 mM HEPES buffer, pH 8.0. Cleaved protein was separated from TEV protease using Ni-NTA affinity chromatography, further purified by size-exclusion chromatography on a HiLoad 16/600 Superdex 200 Prep Grade (GE Healthcare) in standard lysis buffer, and concentrated to ~10 mg mL^−1^.

### Protein structural characterization by small-angle X-ray scattering

Small-angle X-ray Scattering Studies (SAXS) were performed at the BioCAT/18ID beamline at the Advanced Photon Source, Argonne National Laboratory. Each native protein at a concentration of 5 mg mL^−1^ was loaded onto a Superdex 200 column, pre-equilibrated with 20 mM HEPES buffer, pH 8.0, containing 150 mM NaCl and 2 mM TCEP, using a size exclusion chromatographic system (AKTA pure, GE Healthcare) and monodisperse samples were routed directly and continuously into a flow cell for subsequent X-ray exposure. A photon-counting PILATUS 3 1M detector was used to record the scattered X-rays at a wavelength of 1.03 Å. The sample-to-detector distance was 3.5 m and yielded a range of 0.005–0.33 Å^−1^ for the momentum transfer (*q* = 4π sinΘ/λ, where 2Θ is the angle between the incident and scattered beam and λ is the X-ray wavelength). The standard data reduction procedure for biological SAXS was performed with the programs in the ATSAS package (Petoukhov et al., 2012). The radius of gyration Rg and the maximum dimension of the particle D_max_ were obtained using Guinier analysis and the calculated pair distance distribution function p(r). Ten bead models were reconstructed *ab initio* using DAMMIF (Franke and Svergun, 2009) and averaged using DAMAVER (Volkov and Svergun, 2003). Atomic coordinates of P5C reductase from *Streptococcus pyogenes* (PDB 2AMF; Nocek et al., 2005) were superimposed using the program SUBCOMB of ATSAS package (Petoukhov et al., 2012).

### Enzyme assay

The physiological, forward reaction of P5C reductase was measured at 30°C following the oxidation of NAD(P)H at 340 nm. Under standard conditions, the assay mixture contained 20 mM Tris-HCl buffer, pH 7.0, 0.5 mM NADH or NADPH, and 1 mM l-P5C in a final volume of 0.2 mL. Proteins were column buffer-exchanged just before the analysis to remove Hepes buffer, which was replaced with 10 mM Tris-HCl buffer, pH 7.0. dl-P5C was synthesized by the periodate oxidation of δ-*allo*-hydroxylysine (Sigma H0377) and purified by cation-exchange chromatography according to Williams and Frank (1975). A limiting amount of enzyme (about 0.1, 2, 0.4, and 10 μg of the purified ProH, ProI, ProG, and ComER, respectively) was added to the pre-warmed mixture, and the decrease in absorbance at 340 nm was recorded at 30-s intervals and up to 25 min through an optical path of 0.5 cm. Unspecific oxidation of pyridine dinucleotides, measured in parallel blanks in which P5C had been omitted, was subtracted, and activity was calculated from the initial linear rate on the assumption of a molar extinction coefficient for NAD(P)H of 6,220 M^−1^ cm^−1^. Linear regression analysis was computed with Prism 6 (version 6.03, GraphPad Software, Inc., USA). Protein content was determined by the Coomassie Blue method (Bradford, 1976), using bovine serum albumin as the standard. For the purified proteins, direct absorbance at 280 nm was used instead, and the concentration was calculated by a deduced (http://web.expasy.org/protparam/) molar extinction coefficient of 9,065, 10,555, 14,565, and 10,805 M^−1^ cm^−1^ for ProH, ProI, ProG, and ComER, respectively.

### Kinetic analyses

To calculate substrate affinity constants and enzyme maximum rates, non-variable substrates were fixed at the same levels as in standard assay. Depending on the protein, the concentration of l-P5C ranged from 40 to 2,000 μM, the concentration of NADH was varied from 50 to 800 μM and that of NADPH ranged from 12 to 400 μM. To evaluate the occurrence of product inhibition, proline and NAD(P)^+^ were added to the standard assay mixture at increasing levels, ranging from 5 to 500 mM (proline), from 0.02 to 12.5 mM (NADP^+^), or from 0.8 to 25 mM (NAD^+^). To assess the effect of ions on either the NADH- or the NADPH-dependent activity, increasing concentrations of NaCl from 10 to 1,000 mM were added to the reaction mixture. In all cases, the resulting activity was expressed as percent of that in untreated controls. All assays were performed in triplicate. K_M_ and V_max_ values and the concentrations causing 50% inhibition (IC_50_) of P5C reductase activity, as well as their confidence limits, were estimated by non-linear regression analysis using Prism 6. Catalytic constants were calculated from V_max_ values for a single monomer, not taking into account the oligomeric composition of the native holoenzyme.

## Results

### Sequence analysis of ProI, ProH, ProG, and ComER

*Bacillus subtilis* genome contains four genes that can be predicted by homology as possible P5C reductases. Based on the sequence alignment blueprint (Figure 2) outlined in a previous study (Forlani et al., 2015c), the presence of the two conserved cofactor-binding fingerprint motifs B and C was clearly detectable in all four deduced proteins. The motif B, the so-called Rossman fold hallmark motif with the G-x-x-G-x-G-x-M consensus sequence, is conserved in all representatives except ProH, in which the first glycine is replaced by an alanine residue. This motif identifies the presence of the glycine-rich loop, which interacts with the cofactor phosphate moiety. There are other three residues (V–K–P) that are a part of the motif B, and impact the interaction with the pyrophosphate moiety of the cofactor, even though their position is ~60 residues from the hallmark motif (Forlani et al., 2015c). The middle residue (a lysine) is conserved in all P5C reductases except ProG. The motif C forms a conserved loop that contributes to the formation of the hydrophobic region around the cofactor nicotinamide ring. The sequence motif R-x-M-x-N shows a presence of methionine residue, which in concert with the methionine of motif B, surrounds the cofactor's nicotinamide ring. The exact match is found in ProI and ProH, while ProG and ComER appear to have a variant of that motif with sequence R-x-I-x-A/S, the methionine being replaced by another hydrophobic residue, isoleucine. In contrast to the cofactor binding which is positioned at the N-terminal end, the binding loop for P5C (motif E) with the consensus sequence S-P-G-G-T-T (Nocek et al., 2005; Forlani et al., 2015c; Ruszkowski et al., 2015), is positioned at the C-terminal end. The cavity formed by the residues forming this motif has been predicted to play a critical role in binding of the pyrroline ring. Based on the structural data it was deducted that the presence of the proline and glycine residues in the center allows positioning of the flanking serine and threonine residues such that they could interact with the carboxylate moiety of the substrate. Interestingly, there are irregularities in the sequences of the motif in all four putative P5C reductases. While ProI has an exact matching sequence in the motif region, ComER is on the opposite side of the spectrum with only one threonine present. In ComER, valines replaced two other conserved residues. It may indicate that ComER lost elements that allow substrate binding, or could use an alternative mechanism. This is even clearer while comparing motif D sequences. Motifs D were shown to form hinge loops with the sequence of G-S-x-P-A (Forlani et al., 2015c) and contribute bonding to stabilized motif E. The sequence of this motif in ProG and ComER appears to be modified; especially the second residue [a serine that has been shown to form the critical interactions with the active site (motif E threonine)] is missing in ComER.

### All putative P5C reductases from *B. subtilis* are able to catalyze *in vitro* the reduction of P5C to proline, but their kinetic and structural properties suggest that ProI and ComER lost their catalytic efficiency or underwent subfunctionalization

The four *B. subtilis* genes coding putative P5C reductases were cloned into the expression vector pMCSG68 and heterologously expressed in *E. coli*. The presence of N-terminal His_6_-tag and the adoption of a stepwise elution protocol allowed the attainment of substantially homogeneous preparations in a single step, although the heterologous proteins eluted at different imidazole concentrations (data not shown). The presence of the His_6_-tag did not have any apparent effect on the enzymatic activity, as similar specific activity values were obtained before and after the cleavage of the purified proteins with TEV protease. Enzyme preparations were moderately stable; if sterilized by filtration (0.22 μm pore size), more than 80% activity was retained after 2 week-storage at 4°C, but < 20% of the initial activity was evident after 1 month.

In all cases the recombinant enzymes showed the capability of catalyzing the P5C-dependent oxidation of NAD(P)H at neutral pH. However, activity levels strongly differed within the group (Table 2), with ProG and ProH showing the typical values observed for P5C reductases, while ProI and ComER produced detectable levels of P5C reduction only when added to the reaction mixture in microgram amounts. Under standard assay conditions and with NADPH as the electron donor, the resulting specific activity values were about 1,500 and 400 nkat mg^−1^ protein for ProH and ProG, respectively, but only 5 nkat mg^−1^ protein in the case of ProI, and <1 nkat mg^−1^ protein for ComER. NADH was found to serve as a substrate as well, but this caused a generalized 50–75% drop of the catalytic rate.

**Table 2:** Structural and functional properties of the four putative P5C reductases from *Bacillus subtilis*.

	**ProH**	**ProI**	**ProG**	**ComER**
Denatured molecular mass [kDa]^a^	32.03	30.90	30.20	30.24
Native molecular mass [kDa]^b^	62.55	84.70	66.10	31.30
Specific activity _(withNADHasthe co−substrate)_ [nkat (mg protein)^−1^]^c^	410 ± 16	2.2 ± 0.2	95.8 ± 1.7	0.43 ± 0.03
Specific activity _(withNADPHastheco−substrate)_ [nkat (mg protein)^−1^]^d^	1,548 ± 43	4.2 ± 0.1	403 ± 9	0.77 ± 0.03
pH optimum^e^	6.54	6.08; 7.12	6.28; 7.73	<5.80
V_max (NADH)_ [nkat (mg protein)^−1^]^f^	987 ± 60	9.2 ± 1.7	577 ± 130	0.55 ± 0.02
V_max (P5C, withNADHastheco−substrate)_ [nkat (mg protein)^−1^]^f^	nc	3.2 ± 0.2	117 ± 9	1.84 ± 0.73
V_max (NADPH)_ [nkat (mg protein)^−1^]^f^	1,612 ± 45	4.5 ± 0.1	644 ± 24	0.83 ± 0.02
V_max (P5C, withNADPHastheco-substrate)_ [nkat (mg protein)^−1^]^f^	~25,100 ± 15,700	4.4 ± 0.2	580 ± 22	3.00 ± 1.02
K_cat (NADH)_ *per* monomer [s^−1^]^g^	nc	0.28	17	0.06
K_cat (NADPH)_ *per* monomer [s^−1^] ^g^	814	0.14	19	0.09
K_M(app)_ for l-P5C _(NADH)_[μM]^f^	nc	408 ± 66	232 ± 48	3150 ± 1660
K_M(app)_ for l-P5C _(NADPH)_[μM]^f^	~14,500 ± 9,780	203 ± 30	64.9 ± 8.1	3150 ± 1420
K_M(app)_ for NADH [μM] ^f^	786 ± 36	1,810 ± 480	3,010 ± 840	226 ± 14
K_M(app)_ for NADPH [μM] ^f^	13.6 ± 2.6	51.8 ± 2.8	232 ± 18	1.2 ± 0.4
K_cat_/K_M(NADH)_ [M^−1^ s^−1^]	nc	1.5 × 10^2^	5.7 × 10^3^	2.7 × 10^2^
K_cat_/K_M(NADPH)_ [M^−1^ s^−1^]	6.3 × 10^7^	2.6 × 10^3^	8.4 × 10^4^	7.5 × 10^4^

a *Calculated (http://web.expasy.org/protparam/) from the deduced amino acid sequence*.

b *As determined by Small-Angle X-ray Scattering*.

c *Measured under standard assay conditions (1 mM l-P5C, 0.5 mM NADH, pH 7.0)*.

d *Measured under standard assay conditions (1 mM l-P5C, 0.5 mM NADPH, pH 7.0)*.

e *Measured with NADPH as the electron donor*.

f *Determined at pH 7.0 by varying a given substrate, with invariable substrates fixed at 1 mM l-P5C and 0.5 mM NADH or NADPH*.

g *Estimated for a single monomer using the calculated denatured molecular mass*.

*nc, not calculable*.

**Figure 3:**
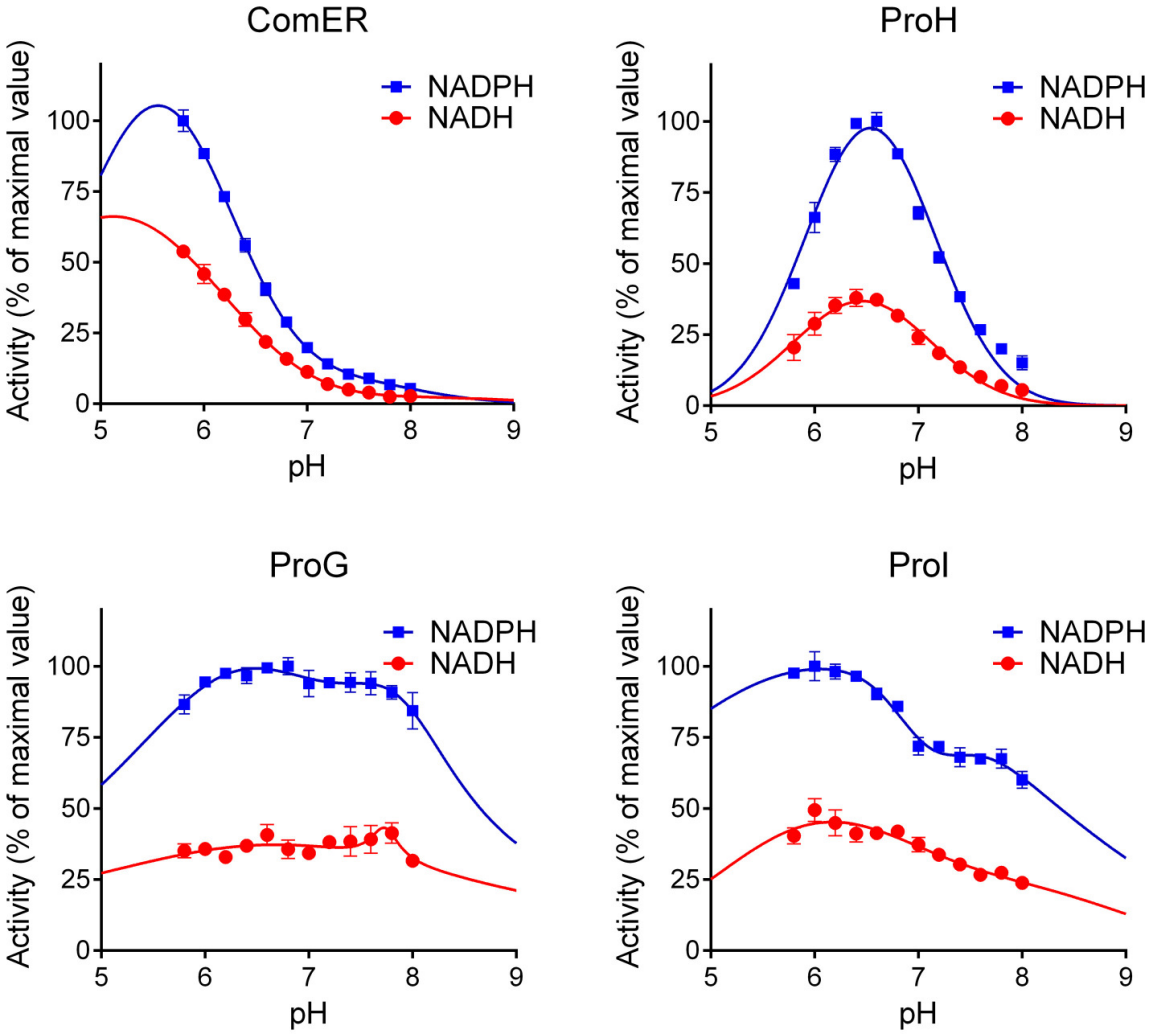
pH-Dependency of the activity of *B. subtilis* P5C reductases. The activity of the four purified proteins was measured with either NADPH or NADH as the electron donor under standard assay conditions at varying the pH of the reaction mixture. To allow a better comparison among the obtained patterns, in each case the activity was expressed by assigning the value 100 to the maximal rate obtained for a given enzyme at varying the pH in the range 5.75–8.00. Results are mean ± SE over three replicates.

**Figure 4:**
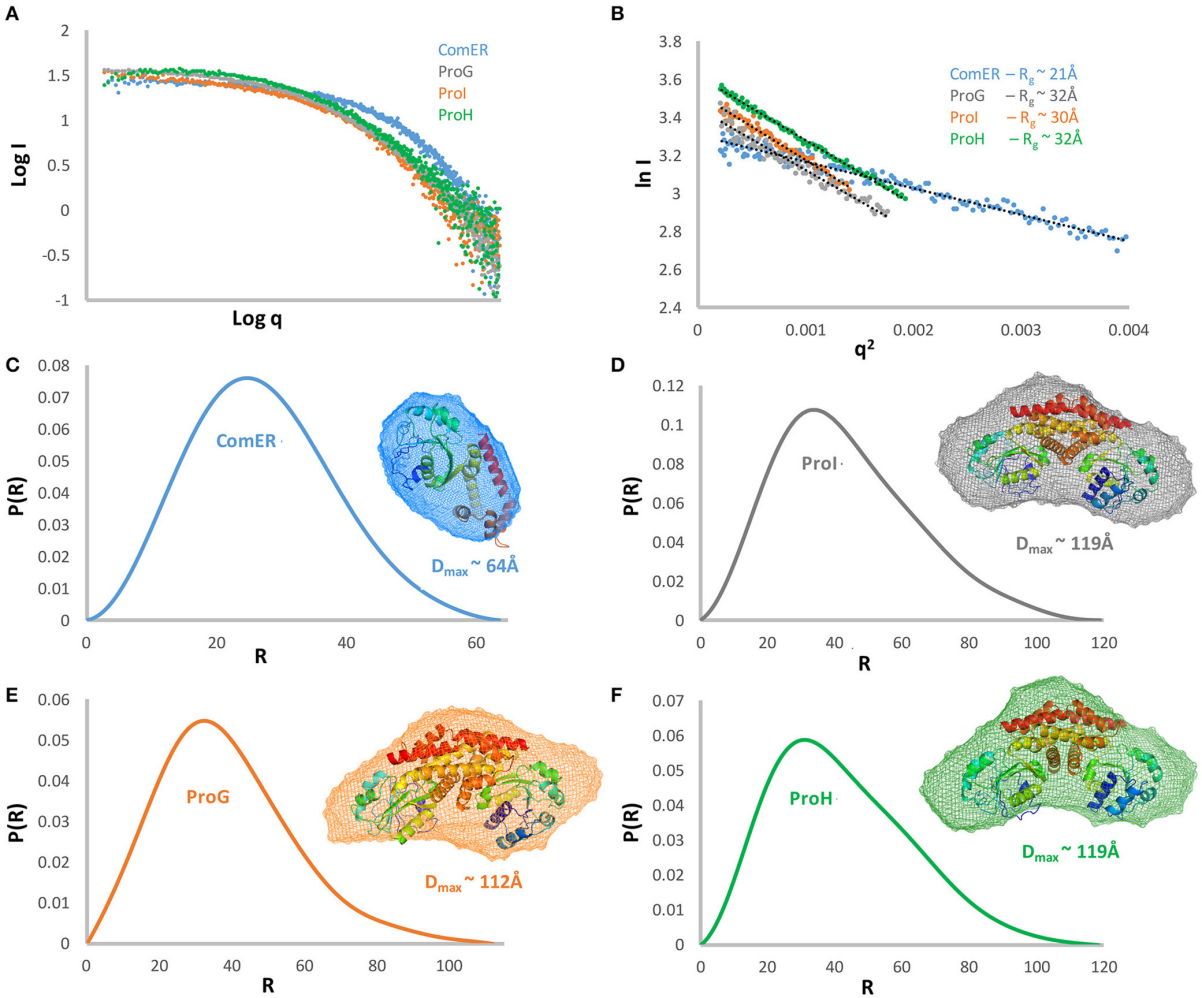
Structural characterization of *B. subtilis* P5C reductases by Small-Angle X-ray Scattering. The experimental scattering curve for each of the proteins is displayed as dots **(A)**. A Guinier plot of the scattering curve is shown with a line of best fit **(B)**. The resulting Rg showed that three proteins have a similar state with R_g_ ~ of 30–32Å and D_max_ ~112 and 119 Å **(D–F)**, while ComER is clearly different with R_g_ ~ 21 Å, and D_max_of 64 Å **(C)**. Pair distance distribution function and modeling of SAXS data showing *ab initio* averaged envelope (transparent mesh reconstruction) was superposed with the ribbon representation of the crystallographic dimer of P5C reductase based on the PDB structure 2AHR. All results concerning the same protein are shown in the same color.

The kinetic characterization of ComER revealed some unusual features for a P5C reductase. The enzyme showed a highest affinity for NADPH, with an apparent K_M_ value of about 1 μM. In contrast, a lowest affinity was observed for l-P5C, with an estimated half-maximal rate at concentrations as high as 3 mM (Table 2). This corresponds to an average value of ~10^5^ M^−1^ s^−1^ of K_cat_/K_M_ with NADPH, but to a strikingly low value with P5C (K_cat_/K_M_ = 29 M^−1^ s^−1^). Taking into account that high levels of P5C are cytotoxic (Deuschle et al., 2004) and that under physiological conditions its intracellular concentration ranges from 10 to 20 μM (Giberti et al., 2017), the resulting activity *in vivo* of ComER would be negligible (~0.01 nkat mg^−1^ protein). Moreover, with NADPH as the electron donor the activity was unaffected by either high proline concentrations exceeding 500 mM, or NaCl levels as high as 1 M (Table 3). When the pH-activity relationship was tested, ComER showed an uncommon pattern in which the catalytic rate was as higher as lower the pH over the entire range tested (5.8–8.0; Figure 3). Additionally, the SAXS results (Figure 4) were consistent with a molecular mass of 33 kDa, clearly pointing to ComER as a monomer. The large-scale analysis of all P5C reductases characterized to date showed that at least a dimeric organization is required to be fully functional (Forlani et al., 2015c). Therefore, these results strongly suggest that ComER may be instead an NADPH-dependent reductase using *in vivo* other aldehyde(s) as the substrate.

**Table 3:** Concentrations of products and salt inhibiting by 50% (IC_50_) the activity of the four putative P5C reductases from *B. subtilis*.

	**ProH**	**ProI**	**ProG**	**ComER**
Proline _(NADPHastheco−substrate)_ [mM]	41.4 ± 4.2	145 ± 19	22.3 ± 2.1	>1,000
Proline _(NADHastheco−substrate)_ [mM]	667 ± 112	232 ± 43	175 ± 17	>1,000
NAD^+^_(NADPHastheco−substrate)_ [mM]	>50	23.6 ± 4.1	>50	24.8 ± 4.5
NAD^+^_(NADHastheco−substrate)_ [mM]	44.5 ± 11.6	6.22 ± 0.60	>50	29.7 ± 3.7
NADP^+^_(NADPHastheco−substrate)_ [mM]	19.0 ± 7.7	4.80 ± 0.62	4.39 ± 1.11	not inhibitory
NADP^+^_(NADHastheco−substrate)_ [mM]	0.66 ± 0.10	0.34 ± 0.04	0.23 ± 0.03	0.48 ± 0.07
NaCl _(NADPHastheco−substrate)_ [mM]	484 ± 43	367 ± 41	745 ± 191	not inhibitory
NaCl _(NADHastheco−substrate)_ [mM]	462 ± 75	208 ± 38	416 ± 78	114

Concerning ProI, more *canonical* properties were found. A higher V_max_ value with NADH than NADPH as the co-factor was paralleled by a 35-fold higher affinity for the latter, resulting under standard assay conditions in a higher specific activity level with NADPH (Table 2). The measurement of the activity as a function of pH showed two optimal values at pH 7.1 and 6.1 (Figure 3), possibly reflecting the intrinsic enzyme property and the equilibrium in solution between P5C and glutamate semialdehyde (which seems to be the actual substrate of P5C-metabolizing enzymes; Arentson et al., 2012; Forlani et al., 2015a), respectively. P5C reduction was inhibited in a concentration-dependent manner by both proline or NaCl (Table 3). However, the activity rate was extremely low, with only 1 catalytic event every 4–7 s (Table 2), and even with the preferred electron donor the corresponding specificity constant was < 3 × 10^3^ M^−1^ s^−1^. If the relatively low affinity constant for P5C (K_M_ values in the 10^−4^ to 10^−3^ M range) is also considered, the contribution of ProI to the overall P5C reductase activity inside the cell may be questionable.

### Results of the functional characterization of the two more active P5C reductases suggest a constitutive function for ProG, whereas ProH features seem consistent with a role in satisfying increased proline demand under stress

The specific activity values found for the other two heterologously-expressed proteins were 200–2,000-fold higher (Table 2), thereby similar to those previously described for single P5C reductases in other eubacteria (e.g., Petrollino and Forlani, 2012). In both cases a higher catalytic rate under standard assay conditions was evident with NADPH as the electron donor, and the Michaelis constant for NADH was 15–60-fold higher than for NADPH (Table 2). Moreover, the presence of NADP^+^ in the 10^−4^–10^−3^ M range progressively inhibited the NADH-dependent activity without substantially affecting that driven by NADPH (Table 3), a result that also in this species strengthens the possibility that NADPH may be the only co-factor *in vivo* (Ruszkowski et al., 2015).

**Figure 5:**
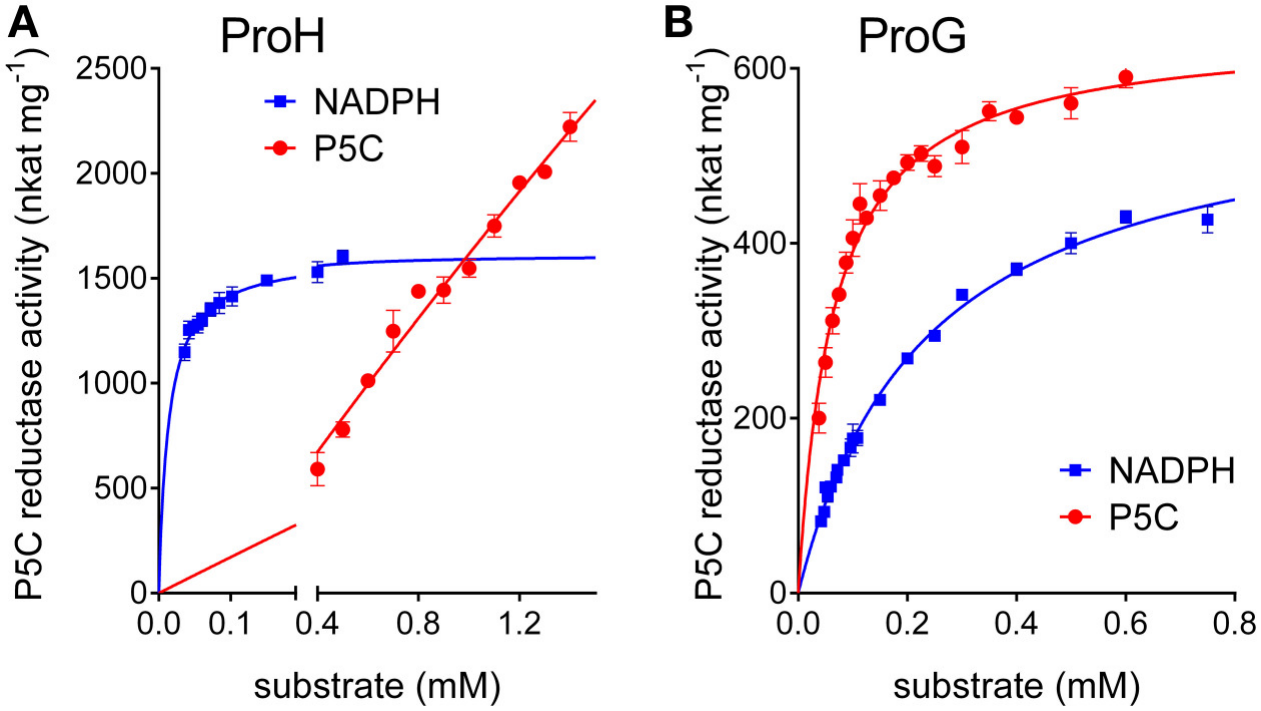
Comparison of substrate affinities between **(A)** ProH and **(B)** ProG. The specific activity of the two functional enzymes was measured with NADPH as the electron donor at varying the concentration of either substrate. The unvariable substrate was fixed as in standard assay. Results are mean ± SE over three replicates.

Interestingly, some relevant differences were found between the two enzymes. Concerning the pH-activity relationship, ProG showed a similar catalytic rate over the whole range tested, whereas for ProH a prominent maximum was evident around pH 6.5 (Figure 3). More remarkably, completely different patterns were found at increasing substrate concentrations. In the case of ProG, saturating conditions were obtained in the 50–500 μM range for both substrates (Figure 5B), with half maximal activity at 70 μM P5C and 240 μM NADPH (Table 2). On the contrary, ProH showed a higher affinity for the pyridine dinucleotide, with more than 80% V_max_ achieved at concentrations as low as 50 μM, but a much lower affinity for the specific substrate, so as the enzyme did not reach saturation even at millimolar P5C concentrations (Figure 5A). As a consequence, ProH showed a highest theorical specificity constant of 6 × 10^7^ M^−1^ s^−1^ vs. the average constant of 8 × 10^4^ M^−1^ s^−1^ for ProG. However, at the physiological P5C concentration of 20 μM the activity rate of the two enzymes would be higher for ProG (about 34 and 148 nkat mg^−1^ protein for ProH and ProG, respectively). Yet, following the increase of the intracellular level of P5C, the activity of ProH would increase much more than that of ProG, with similar values (about 550 nkat mg^−1^ protein) at 330 μM. With a further increase of P5C level to 500 μM, the activity of ProG would be only slightly enhanced (about 580 nkat mg^−1^ protein), whereas that of ProH would be further stimulated by 50% (835 nkat mg^−1^ protein).

**Figure 6:**
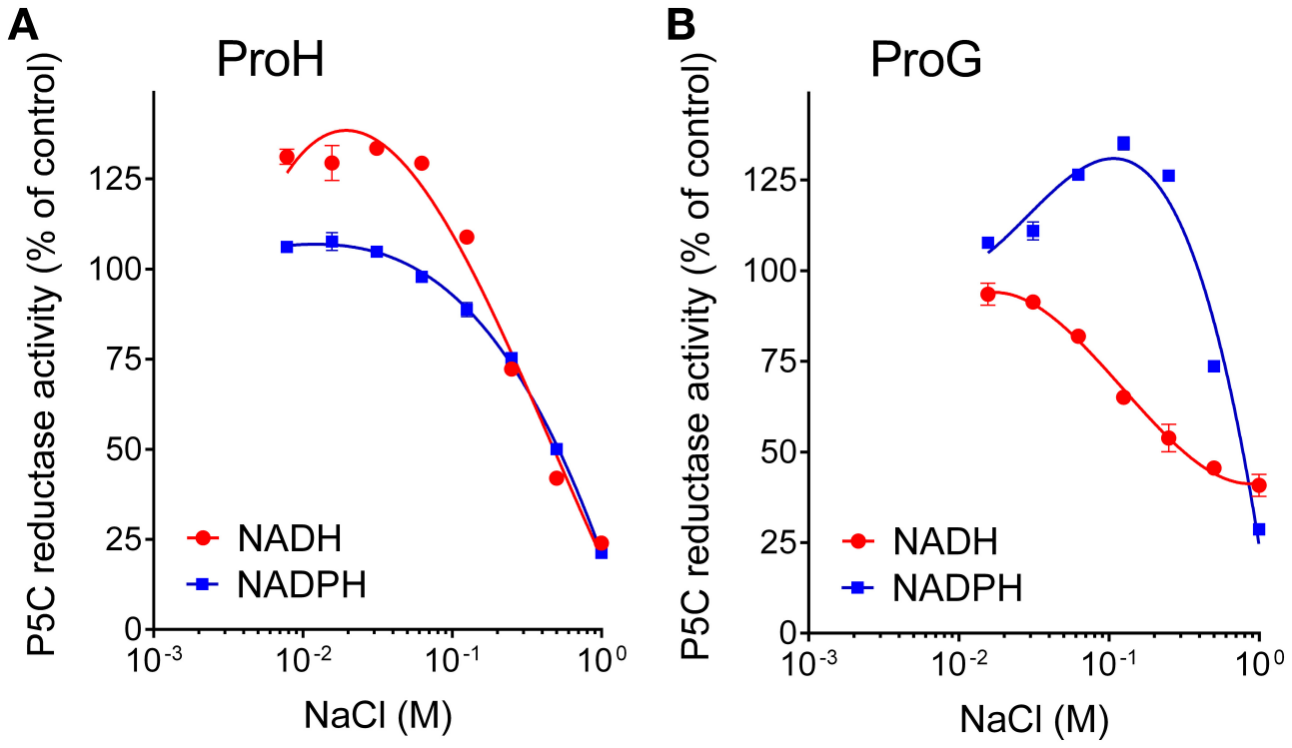
Effect of increasing concentrations of salt on the activity of **(A)** ProH and **(B)** ProG. Enzyme activity was measured with either NADPH or NADH as the electron donor under standard assay conditions by adding increasing levels of NaCl to the reaction mixture. Activity values were then expressed as percent of values obtained with controls in which no NaCl had been added to the reaction mixture. Results are mean ± SE over three replicates.

Concerning mechanisms for post-translational regulation, both enzymes were subjected to product inhibition, being proline concentrations in the range 10–100 mM able to inhibit their activity (Table 3), with IC_50_ values slightly but significantly different. A more remarkable difference was found with respect to salt. Not taking into account the NADH-dependent activity, which seems of marginal importance *in vivo*, a noteworthy dissimilar pattern was found by adding to the assay mixture increasing concentrations of NaCl in a physiological range (10–100 mM). In the case of ProH the addition was substantially ineffective, and at doses exceeding 100 mM enzyme inhibition took place (Figure 6A). On the contrary the activity of ProG was progressively enhanced by up to 35%, and only over 200 mM the presence of salt became inhibitory (Figure 6B). On the whole, all their features would make ProG more suitable for non-stressful conditions, as its activity is not influenced by pH fluctuations, reaches about 25% of maximal level at the physiologically lowest concentrations of P5C, and may be immediately enhanced by an increase of salt level in the cell. On the contrary, ProH would meet cell requirement under stress, since its activity increases almost linearly as a function of P5C concentration allowing the attainment of a higher rate of proline synthesis, is not significantly influenced by salt concentration, and is further stimulated by cytoplasmic acidification.

## Discussion

In the current study, the putative P5C reductases from *B. subtilis* were successfully expressed and purified allowing biochemical characterization and structural studies. The SAXS experiments revealed that the four proteins could be divided into two groups based on their low-resolution structures. The first group contains three dimeric representatives in solution with maximum molecular dimensions and radius of gyration of (119Å; Rg ~ 30Å) for ProI, (119Å Rg ~32Å) for ProH, and (112Å; Rg ~ 30Å) for ProG, respectively. Their envelopes have triangle shapes, which well fit the dimer of the previously determined high-resolution structure of the orthologue from *S. pyogenes* (Figure 4; Nocek et al., 2005). In contrast, ComER is the single representative of the second group, as it forms monomer in solution with a maximum molecular dimension of ~64 Å and radius of gyration of ~21Å. The low-resolution envelope has a globular shape able to fit only a single chain of the P5C reductase orthologue. We previously reported that the dimeric arrangement is a prerequisite for a proper catalysis of P5C reduction (Forlani et al., 2015c).

The kinetic analysis further supports a functional divergence for ComER, and suggests subfunctionalization. Although the enzyme was still found able to catalyze P5C reduction *in vitro*, its properties indicate that the activity would be negligible *in vivo*, with a sharp reduction in substrate affinity. A significant catalytic rate would require millimolar intracellular concentrations of P5C, a condition that would be lethal due to its cytotoxicity (Deuschle et al., 2004; Giberti et al., 2017). It is also consistent with early findings showing that the simultaneous defect of ProG, ProI, and ProH, but not of ComER, was required to confer proline auxotrophy (Belitsky et al., 2001). ComER properties could be the consequence of an increasing number of subsequent mutations causing the loss of the ability to form dimers and a decrease in its catalytic efficiency. However, the enzyme showed a highest apparent affinity for NADPH, greater than that of recently characterized plant and bacterial P5C reductases (Petrollino and Forlani, 2012; Giberti et al., 2014; Forlani et al., 2015b). Moreover, recent results are suggestive of a different physiological function for ComER. Indeed, in two *Bacillus* species mutations in the comER gene were found to cause defects in biofilm formation and a delay in spore development, suggesting that comER may act as an early regulator in the complex regulatory circuit that controls the transition from planktonic growth to sporulation (Yan et al., 2016). Also, our data showed increasing activity at decreasing pH values (Figure 3), and it is well-known that during *Bacillus* sporulation the pH of the forespore falls by at least one unit (Magill et al., 1994). Therefore, it seems that ComER may most likely play a role as a Rossmann-type reductase specific for another, yet unknown substrate. Consistently, proline does not exert any inhibitory effect on its activity (Table 3).

In regards to the representatives of the first group, the functional properties of ProI are consistent with those of previously studied P5C reductases, although the catalytic constant appears low. The apparent affinities for both substrates are similar to those calculated for most enzyme representatives (25 μM < X < 75 μM and 200 μM < Y < 500 μM for NADPH and P5C, respectively; Petrollino and Forlani, 2012; Forlani et al., 2015b; Ruszkowski et al., 2015). Also, the biphasic profile of activity as a function of pH (Figure 3) is typical of P5C-metabolizing enzymes, with the maximum around pH 6.3 a consequence of the equilibrium in solution between P5C and glutamate semialdehyde, which was proposed as the actual substrate (Arentson et al., 2012). Moreover, ProI activity was found to be subjected to product inhibition by both proline and NADP^+^, in a range of concentrations similar to those affecting the other two, more active isoforms (Table 2). Finally, the protein exists in solution as a homodimer (Figure 4), a minimal structural feature of P5C reductases. The overall picture seems to point at an enzyme in which some deleterious mutations have reduced its catalytic efficiency, but which has not undergone subfunctionalization. Indeed, based on kinetic measurements, even at the low physiological levels of the two substrates ProI would retain a specific activity of about 1 nkat mg^−1^. The possibility that the enzyme catalyzes P5C reduction at significant rates *in vivo* is also supported by early data showing that only a triple mutation also affecting ProI (besides ProH and ProG) causes proline auxotrophy in *B. subtilis* (Belitsky et al., 2001).

The biochemical and structural features of the two other representatives, namely ProG and ProH, were also typical of fully functional P5C reductases: a homodimeric composition (Figure 4), a higher affinity for NADPH than for NADH, affinity constants in the micromolar range (Table 2). Specific activity levels *in vitro* were significantly lower than those found under the same experimental conditions for the plant enzyme (Giberti et al., 2014; Forlani et al., 2015b; Ruszkowski et al., 2015), but similar to those of representatives from other bacterial sources (Petrollino and Forlani, 2012). Notably, in both cases, the activity was found inhibited by enzyme products, with a progressive reduction of the catalytic rate in the presence of proline concentrations in the range from 10 to 100 mM (Table 3). However, some significant differences were also evident between the two enzyme forms, which can shed some light on a possibly distinct physiological role. In the case of ProG, a lowest apparent affinity constant for P5C and a relatively high K_*M*_ for NADPH would allow the attainment of a significant catalytic rate at the low concentrations of the substrate in the cell under non-stressful conditions (Deuschle et al., 2004; Giberti et al., 2017), and a modulation of enzyme activity as a function of fluctuations of the adenylate or redox status of the cell (Hare and Cress, 1997; Giberti et al., 2014). Moreover, maximal activity was retained over a broad pH range (6–8; Figure 3), and an IC_50_ for proline of about 20 mM would result *in vivo* in a low homeostatic level of this imino acid. Overall, these features seem compatible with a role of ProG in providing proline for growth, as a building block for proteins. Consistently, the proI proG double mutant showed an extended lag period before initiation of growth in the absence of exogenous proline (Belitsky et al., 2001).

Conversely, ProH showed a higher affinity for NADPH, but also an even higher apparent K_M_ for P5C, one that hampered the attainment of saturating conditions *in vitro* (Table 2). Such features imply that, although the calculated V_max_ for ProH is about 50-fold higher than that of ProG, at physiological substrate levels the specific activity of the two enzymes would be similar. On the other hand, the high K_M_ for P5C (Figure 5) causes that the activity of ProH would immediately respond to any increase of specific substrate levels with an increased catalytic rate, allowing the maintenance of the potentially toxic P5C at low concentrations. Moreover, the slightly but significantly higher IC_50_ for proline (Table 3) would result in higher homeostatic levels of the free imino acid as a consequence of ProH expression than (or besides that) of ProG. Finally, the profile of activity as a function of pH (Figure 3) showed a striking increase in the catalytic rate at decreasing the pH from 7.5 to 6.5, and cytoplasmic acidification has been reported as a possible consequence of hyperosmotic treatments (Katsuhara et al., 1989). All of these properties would fit with the need to synthesize and maintain increased levels of free proline without the risk of a concomitant increase of P5C concentration. All features seem to consistently point out a role for ProH as the enzyme specifically involved in the osmo-induced synthesis of proline. Our results strengthened previous genetic data findings showing induction of the *proHJ* locus by high concentrations of salt (unpublished data in Belitsky et al., 2001), as well as those of deletion analysis of the *proHJ* promoter region showing a 126-bp DNA segment carrying all sequences required in *cis* for osmoregulated transcription (Brill et al., 2011).

In summary, biophysical and biochemical data provided in this study suggest that only two P5C reductases contribute to proline synthesis in *B. subtilis*, where ProG ensures the building blocks for protein synthesis and ProH allows increased production of proline as a compatible solute. Conversely, ProI seems a dysfunctional gene product, whereas ComER most likely represents a Rossmann-type reductase specific for other physiological substrate(s), derived from a duplicated P5C reductase gene that underwent subfunctionalization.

## Author contributions

GF and BN designed the study, BN, GF, and SC collected the data, GF, BN, SC, and AJ performed the analysis and data interpretation, and GF and BN wrote the manuscript with critical input by all authors.

### Conflict of interest statement

The authors declare that the research was conducted in the absence of any commercial or financial relationships that could be construed as a potential conflict of interest.
